# MA-CharNet: Multi-angle fusion character recognition network

**DOI:** 10.1371/journal.pone.0272601

**Published:** 2022-08-29

**Authors:** Qingyu Wang, Jing Liu, Ziqi Zhu, Chunhua Deng

**Affiliations:** 1 School of Computer Science and Technology, Wuhan University of Science and Technology, Wuhan, China; 2 Hubei Province Key Laboratory of Intelligent Information Processing and Real-time Industrial System, Wuhan, China; University of Engineering and Technology Taxila Pakistan, PAKISTAN

## Abstract

Irregular text recognition of natural scene is a challenging task due to large span of character angles and morphological diversity of a word. Recent work first rectifies curved word region, and then employ sequence algorithm to complete the recognition task. However, this strategy largely depends on rectification quality of the text region, and cannot be applied to large difference between tilt angles of character. In this work, a novel anchor-free network structure of rotating character detection is proposed, which includes multiple sub-angle domain branch networks, and the corresponding branch network can be selected adaptively according to character tilt angle. Meanwhile, a curvature Adaptive Text linking method is proposed to connect the discrete strings detected on the two-dimensional plane into words according to people’s habits. We achieved state-of-the-art performance on two irregular texts (TotalText, CTW1500), outperforming state-of-the-art by 2.4% and 2.7%, respectively. The experimental results demonstrate the effectiveness of the proposed algorithm.

## 1 Introduction

In recent years, numeral recognition [1, 2] and character recognition in natural scenes have attracted increasing attention and their application has been widely used, such as robot navigation [3] and image retrieval [4]. With the vigorous promotion of deep learning [5], scene text recognition has made rapid progress [6–11]. However, scene text recognition is still a task with many challenges due to the different text forms in natural scenes (e.g., irregular text layout, diversity of colors, fonts, etc.) and complex background interference.

At present, natural scene text recognition can be roughly divided into two categories: encode-decode based method [6, 12–14] and character detection based method [15, 16]. Encode-decode based method treats words or text sequences as base unit. Its main idea is to convert text detection in two-dimensional images into one-dimensional text recognition and location, which extremely depends on the accuracy of word region segmentation [17, 18]. Therefore, the encode-decode method has some limitations on solving the recognition of curve text sequences. In addition, methods based on sequence are limited to languages based on Latin characters, which is difficult to be extended to non Latin character languages (such as Chinese, Japanese, Korean, etc.).

**Fig 1:**
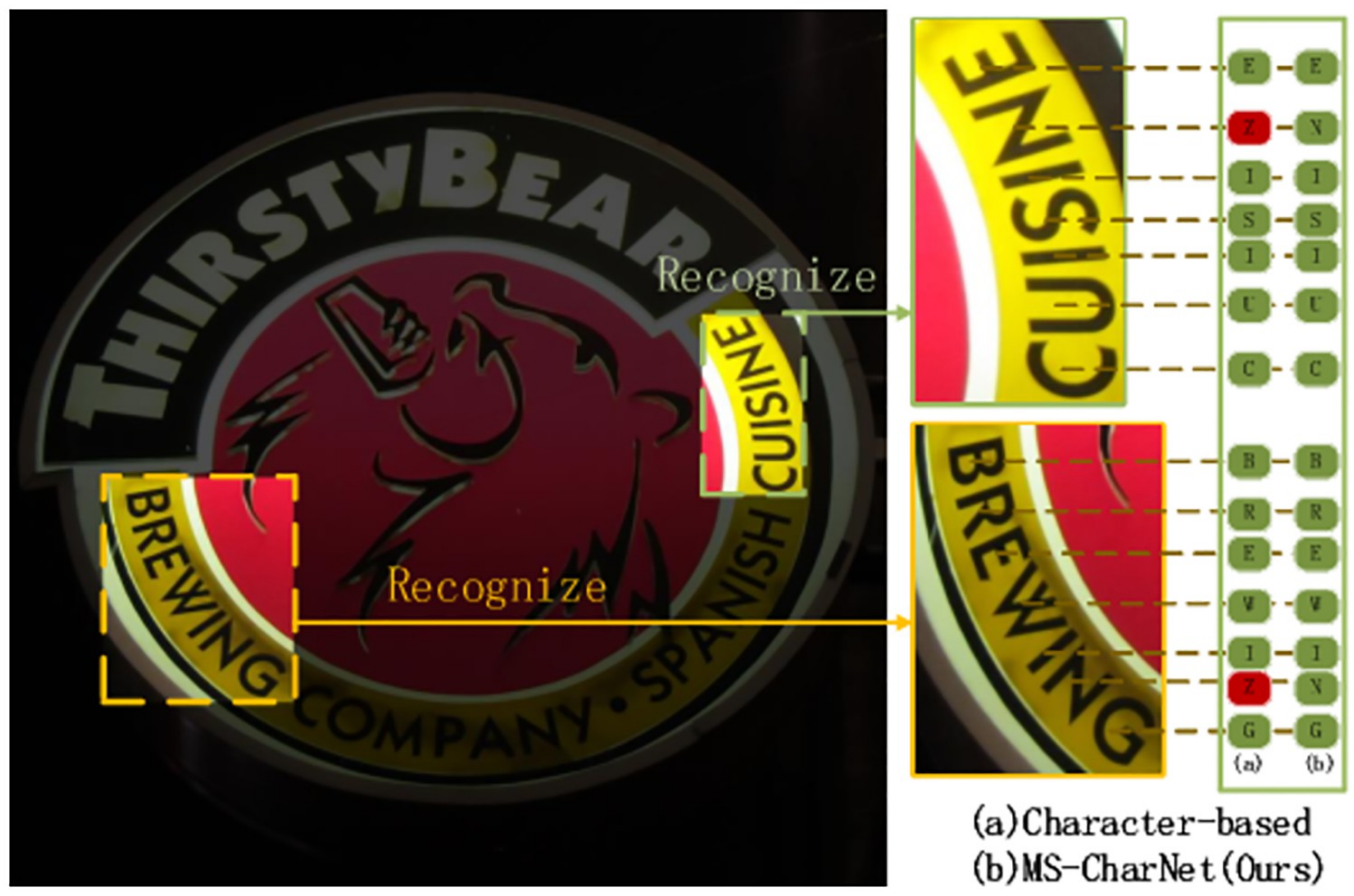
Illustrations of different Char recognition method. When the rotation angles of characters in natural scenes is particularly large, some characters will be extremely similar(such as ‘N’ and ‘Z’). Ordinary character detection methods(a) can hardly cope with this situation, resulting in false detection of characters, which can be well solved by our (b) MS-CharNet’s fusing the character features of each angle domain. Reprinted from [19] under a CC BY license, with permission from IJDAR, original copyright 2020.

Character detection based method generally locates the position of each character, then gives the classification of that character, and finally combines all characters into a string sequence. Character detection is more difficult than object detection due to the diversity of the same character morphology, high similarity between different characters (such as rotated ‘Z’ and ‘N’, ‘6’ and ‘9’, ‘b’ and ‘p’, etc.). and the currently available character detection algorithms [8] cannot cope with this challenge in the case of characters with widely varying tilt angles(Fig 1A).

In order to address above problems, this study divides the rotation angle range of characters into multiple sub-domains, and then learns the character features on the respective sub-domains separately using independent networks. However, there are two problems with using the above strategy directly: (1)The multi-detection networks consume scores of memory or video memory resources and needs a large amount of computation when predicting characters of a large angle span; (2)In the process of inference, the detection network needs to be manually selected according to each character’s rotation angle. In order to solve the problem of large memory or video memory resource consumption(and manual select), this study designs a multi-branch detection network, which corresponds to a sub dataset with small span. In order to solve the problem of correspondence between character angles and sub networks in reasoning process, an adaptive multi branch network is designed in this study. This network has the same performance as the combination of multiple detection networks, but there is no need for manual angle selection or intervention.

Since our method is character-based, it outputs only discrete character detection results. And individual characters lose the semantic information of the original word or text line. In fact, the connection of recognized characters is an essential part of all character recognition methods. Therefore, a method of character connection is needed to complete the task of word recognition on basis of character detection. Most of the existing character connection methods are based on a hypothesis: from left to right or from top to bottom. However, the text in complex natural scenes is multi-directional, and it is easy to reverse in some words with large rotation angle using the above assumptions. Some studies takes the connection between characters as features to learn [20], but it is easy to be disturbed by the noise in a text picture, which also increases additional computation. In this paper, a new character combination method (VDLink) is proposed by using the relationship between the connection curvature of characters and the text direction, which can be adapt to the text with arbitrary arrangement.

The contribution of this paper mainly includes three aspects:

1. A character detection network with adaptive angle selection is proposed, which effectively solves the problem that the shape of the same character is difficult to converge due to the large rotation angle span of the character, and the shape of different characters is similar and difficult to distinguish.
2. A new character combination method (VDLink) is proposed, which can efficiently complete character combination task after detection.

The rest paper is organized as follows: Sec.2 reviews the relevant methods; for the methodology, we describe MA-CharNet in Sec.3. The experiments are discussed and analyzed in Sec.4; the conclusion and the future work are summarized in Sec.5.

## 2 Related work

The main task of scene text recognition is to recognize detected text sequences or edited text images. With the promotion of deep learning, the research has made great progress, gradually moving from the initial recognition of regular text to more challenging areas such as STR (Scene Text Recognize). The current research on Scene Text Recognition can be broadly categorized as follows:

### 2.1 Encode-Decode based methods

Most of the current work uses the Encode-Decode structure for text recognition, which treats the whole text line as a whole and directly maps the input text image to a string sequence. The processing flow of this method is generally divided into four steps: image pre-processing, feature extraction, sequence modeling and sequence transcription. Image preprocessing is used to improve the quality of the image to increase the recognition accuracy. Common image preprocessing methods include super-resolution [23], irregular correction [24], background erasure [25] etc. Feature extraction networks mostly use common deep learning feature extractors and their variants [26, 27], which are used to extract high-level features expressing text; sequence modeling is mainly used to establish contextual relationships between characters, and bidirectional long and short-term memory networks [28] have been applied as mainstream modeling methods in most studies, but it is prone to the problems of gradient disappearance and gradient explosion. In recent years, some new sequence modeling methods can solve the above problems well and gradually gain recognition in the industry, such as sliding window [29], attention [30] etc. The last step of the method, transcription of sequences, is the main challenge, and the two mainstream methods for this step are CTC series methods and the Attention-based methods. Inspired by the successful application of CTC in language recognition and other fields [31] applied CTC to natural scene text recognition for the first time, which significantly improved the recognition performance. Since then, a large number of network methods based on CTC and its variants have been proposed, all of which have shown their powerful decoding performance [12, 32]. Although CTC has good decoding performance, it is difficult to be directly applied to two-dimensional irregular text recognition due to its temporally continuous structural characteristics. The Attention-based approach effectively bridges the difference between regular and irregular text by highlighting the features of the location of characters, and shows obvious superiority in the recognition of irregular text [33].

The application scenarios of the series of methods based on Encode-Decode are limited to the Latin languages, and it is difficult to be applied to non-Latin languages. Moreover, this series of methods strongly depend on the quality of the text correction module and cannot be adapted to the situation where the character skew angle spans a wide range.

### 2.2 Character-based recognition methods

Character-based recognition has been relatively little studied due to the difficulty of obtaining character-level labels, but some classical and effective methods have emerged [34, 35]. The idea of this kind of method is to train the segmentation map to locate the location of characters and then use the character classifier to classify the localized result. Wang *et al*. [36] was the first to train a model using the fraction and location of characters as input and use dictionary matching to get the final prediction, and its performance set the benchmark for research in STR. Driven by deep learning [37], combined convolutional neural networks and unsupervised learning to alleviate the difficulty of obtaining character labels and also achieved good recognition performance. To further improve the recognition ability of the model for characters, some researchers proposed that the characteristics of characters should be learned to distinguish character domains from general objects. Phan *et al*. [34] used SIFT(scale invariant feature transform) descriptors as learning features to significantly improve the performance of character recognition. After that, Yao *et al*. [35] used the stroke information of characters to extract text features, Gordo *et al*. [38] used local mid-level features suitable for building word image representations. Experiments show that such methods are significantly better than the Encode-Decode method in terms of recognition performance and generalization ability. However, it requires accurate character segmentation results. For dense text, it is easy to have adjacent characters stick together. Therefore, the segmentation-based character recognition methods are strongly dependent on and limited by the performance of character segmentation.

We propose to use Anchor-free structure to directly regress the location and type information of characters to cope with the case of dense text sticking. In addition, to cope with the large span of character rotation angles in natural scene texts, we learn the angular properties of characters in addition to the task of character localization and classification.

Before this research, Xing *et al*. [39] took the lead in predicting the geometric information such as location, aspect, and angle of characters using CNN to achieve localization and recognition of characters. However, the angle information it learns is only for character refinement localization, and does not solve the problem of recognizing characters with large span of rotation angles.

Therefore, we divide the rotated characters into multiple domains by angle, and each domain is trained with a different network respectively. The angle information of the learned characters is used to select the corresponding sub-networks for recognition, and the whole rotation angle domain is divided into multiple small rotation angle domains, which solves the problem of rich diversity of the same characters and similarity of different characters due to large rotation angles. Finally, a unified framework is used to fuse the character features learned from each sub-angle domain, which can effectively detect irregular text, especially with better robustness for characters with particularly wide span of rotation angles.

We conduct a comprehensive comparison of advantages and limitations of these methods(Table 1) in terms of the following properties: the basic unit of processing, whether a post-processing algorithm is required to link characters, whether it can recognize curve text, whether it can recognize extreme tilted text, and whether it can be easily applied to non-Latin language.

**Table 1:** Comparison of some representative text recognition methods.

Algorithm	Brief Methodology	Basic unit	Post-algorithmic combination of characters?	Curve text	Extreme tilted text	Easy to apply to non-Latin?
CTC [6]	BiLSTM + Transcription	word	No	✘	✘	No
2D-CTC [13]	2D-CTC + Prediction Alignment	word	No	✔	✘	No
ASTER [21]	RCNN + Attention	word	No	✔	✘	No
SRN [22]	PVA + GSRM + VSFD	word	No	✔	✘	No
SARv2 [14]	LSTM+Attention	word	No	✔	✘	No
CharNet [8]	Detect Char + Grouping	char	Yes	✔	✘	No
MA-CharNet(ours)	Angle Selector + VDLink	char	Yes	✔	✔	Yes

## 3 Proposed method

**Fig 2:**
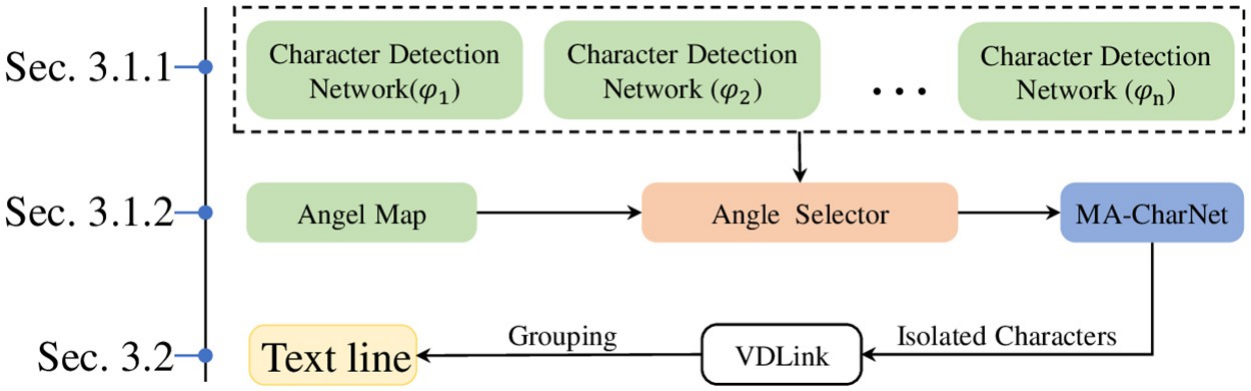
Flowchart of the method section.

In this work, a text recognition method based on character detection is designed for curved text, and it can particularly deal with the situation where the characters have a large tilt angle. Firstly, anchor free network of high positioning accuracy is selected as the backbone network of character detection, and we have added a character angle perception module. On this basis, a multi detection module which can adaptively select branches according to the character tilt angle is designed. The module is equivalent to the combination of multiple detection networks spanning smaller sub-angle domain, yet with a significantly lower computational resource overhead. From a macroscopic point of view, it looks like a network that fuses the character features of each angle domain, so we also refer to the proposed network as MA-CharNet(Multi-angle Fusion Character Recognition Network). Meanwhile, a matching two-dimensional plane discrete character combination method VDLink is designed. The logical relationship between the modules and the guide diagram of this chapter is shown in Fig 2.

### 3.1 MA-CharNet

#### 3.1.1 Character detection network

The character detection network of this work adopts the structure of CenterNet [40], CenterNet learns about the center of a general object, its length and width, and the modification of its length and width properties. On this basis, we add the task of regression character Angle to provide a basis for adaptive selection of subnetworks.

The backbone network of MA-CharNet uses ResNet101 [27], and the convolutional feature map of 1/4 downsampling is used as the input for the subsequent tasks. The design details of each detection head are as follows:

- *H*^*hm*^ predicts the category of characters, the shape of the output feature map is *N*_*s*_ * *w* * *h*, *N*_*s*_ is the number of categories of characters. In this study, *N*_*s*_ is set to 64, to represent 63 characters(52 uppercase and lowercase characters, 10 digitals, one other symbol)and one irrelevant background.
- *H*^*wh*^ predicts the length and width attributes of the characters, and the size of the output feature map is 2 * *w* * *h*, which are used to represent the length and width of characters, respectively.
- *H*^*reg*^ egression correction for the length and width of the characters, whose output feature maps are still of size 2 * *w* * *h*, are used to correct the *H*^*wh*^ predicted length and width attributes, respectively.
- *H*^*ang*^ predicts the angle of the character, and the output shape is 1 * *w* * *h*, which directly regresses the angular value of each angle domain in the feature map. Since this study only uses the angle of the character as the control information for selecting each sub-network, the prediction of the angle does not need to be very precise, as long as it can ensure that the predicted angle falls correctly in the angle interval of the corresponding subangle field network.

It should be noted that the above *H*^*hm*^, *H*^*wh*^, *H*^*reg*^ are all using the structure from CenterNet.

As mentioned above, the character recognition network includes multiple tasks, so the loss function of this model is defined as:

$$\begin{array}{c} L=\alpha L_{hm}+\beta L_{wh}+\gamma L_{reg}+\delta L_{ang}. \end{array}$$ (1)

Since the angle is a continuous value, we use a smoother loss function Smooth L1 loss:

$$\begin{array}{c} L_{ang}=\frac{1}{N}\sum_{i}^{N}\{\begin{array}{cc} 0.5*{\left(\theta_{i}-\hat{\theta_{i}}\right)}^{2} & if\;|\theta_{i}-\hat{\theta_{i}}|<1,\\ |\theta_{i}-\hat{\theta_{i}}|-0.5 & otherwise, \end{array} \end{array}$$ (2)

where *θ*_*i*_ and $\hat{\theta_{i}}$ denote the true angle of the character and the angle value predicted by the global network, respectively.

MA-CharNet is actually a combination of *N* + 1 networks. Denote the rotation angle range of the character as *φ*, and the global network is first trained in this global angle domain, which is to learn the common features of the character and the angle features of the character. Then the angle domain *φ* is divided into *N* sub-domains, and each sub-domain corresponds to an angle domain *φ*_*i*_. Use independent sub-networks to train on each sub-angle domain separately. These *N* + 1 networks are all structured as above, but only global network containing *H*^*ang*^. The sub-networks corresponding to the *N* sub-angle domains share the backbone weights of the global network by loading and freezing the weights of the global network when training these N sub-networks, and updating only multi-heads, i.e. *H*^*hm*^, *H*^*wh*^, *H*^*reg*^.

Denote *φ* as (*ζ*, *η*), where the corresponding angle domain *φ*_*i*_ of each sub-network is related to the global angle domain *φ* as:

$$\begin{array}{c} |\varphi |=\eta -\zeta ,(\eta >\zeta ), \end{array}$$ (3)

where |*φ*| is the angular span value of *φ*. Then the angle domain corresponding to each sub-domain *φ*_*i*_ is:

$$\begin{array}{c} \varphi_{i}=(\frac{i-1}{N}|\varphi |-\Delta \vartheta ,\frac{i}{N}|\varphi |+\Delta \vartheta ), \end{array}$$ (4)

where *i* = 1, 2, ⋯, *N*, to prevent angular edge effects, we set up cross sections for each domain, i.e. Δ*ϑ*.

For subsequent presentation, we denote the network corresponding to the angle domain *φ*_*i*_ as *N*_*i*_, and *H*_*i*_ refers to the feature map output by the detection head of this network(i.e. *H*^*hm*^, *H*^*wh*^, *H*^*reg*^). In particular, the network corresponding to the global angle domain is labeled as *N*_*global*_.

#### 3.1.2 Angle selector

**Fig 3:**
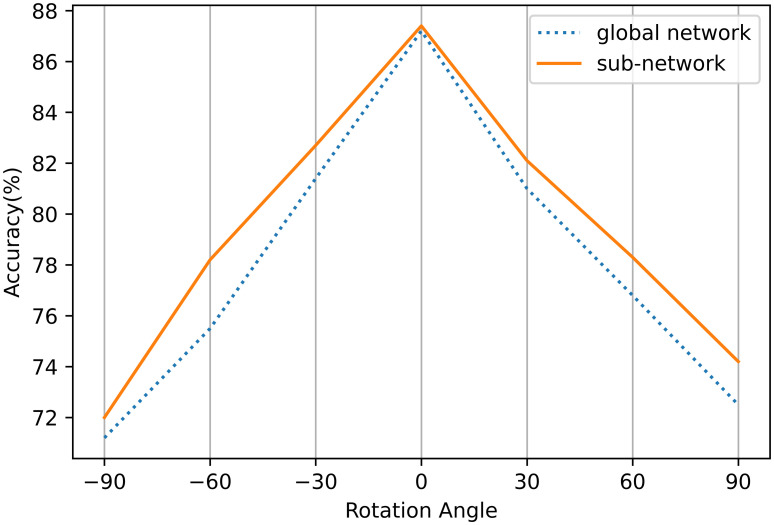
Recognition results on our synth dataset with different rotation angles.

Inspired by FPN(Feature pyramid networks) [41], FPN fuses multiple features of different scale sizes together to solve the multi-scale problems. In this study, the character features located in different angle domains are merged together to solve the problems caused by large rotation span. We experimentally demonstrate(Fig 3) that the recognition performance of the network trained with sub-domains is higher than that of the network trained globally. The angles of the characters on the natural scene pictures cannot be all distributed in the same sub-angle domain, and a specific network cannot be used to complete the recognition task. This requires running multiple sub-networks simultaneously and then synthesizing these results. But this process is strongly dependent on manual work.

**Fig 4:**
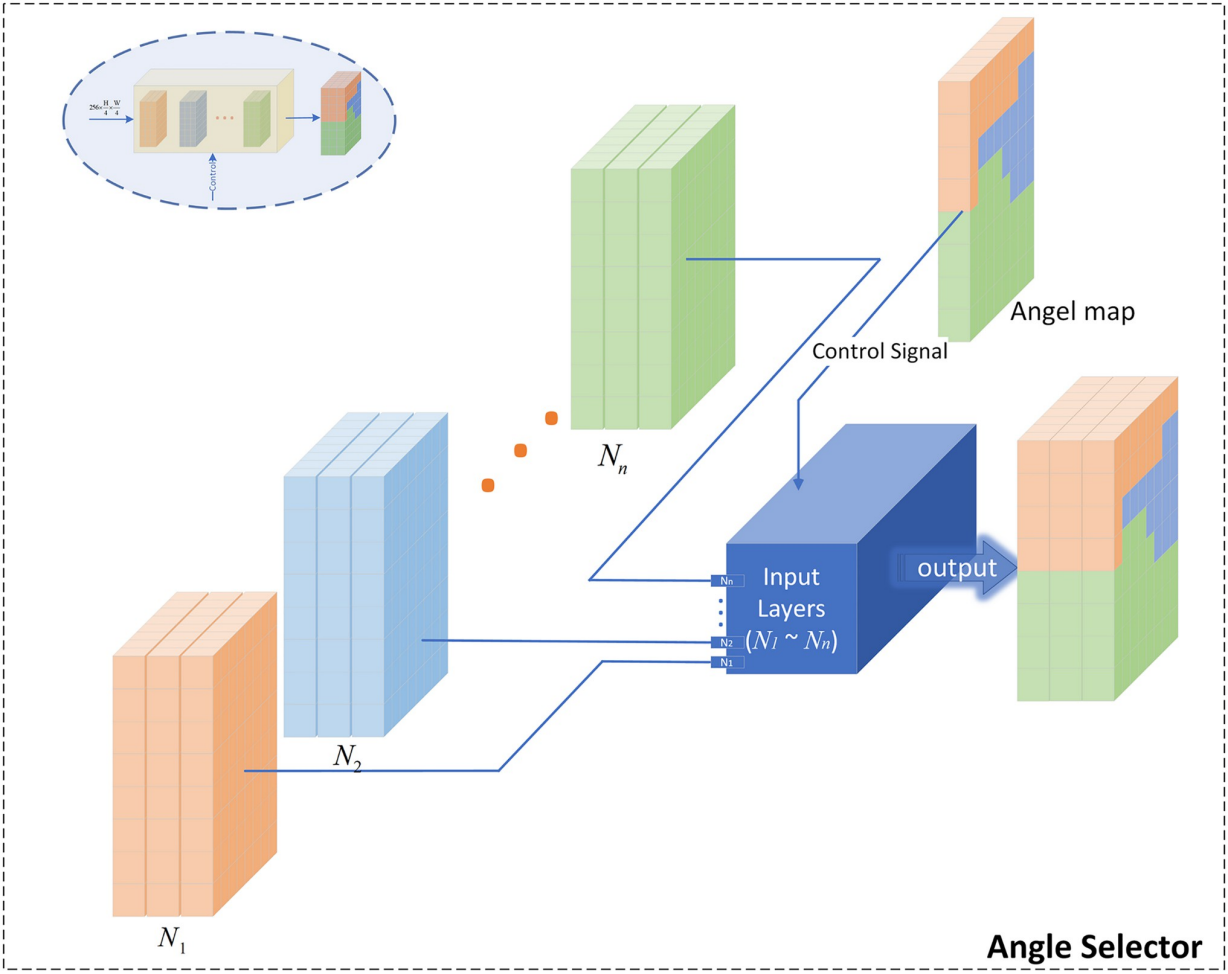
The framework of angle selector. It takes the output of the sub-network of N as input, and the angle prediction map of the global network as control information. It automatically complete the selection of the sub-network through the angle, thus eliminating the need for tedious manual selection.

In order to avoid tedious manual selection, this study designed the Angel Selector to automatically select different sub-networks according to the angle of characters. Its input is the output of the multi-heads of each sub-network prediction feature map, and the control information is the angle prediction map generated by the global network (i.e., $H_{global}^{ang}$), whose structure is shown in Fig 4. In order for each character in the same image to be assigned to the correct sub-network for recognition, it is required that the designed angle selector should be pixel-level. Therefore, we design Angle Selector to first generate angle selection distribution *Mask*_*i*_ for each angle domain network based on the angle prediction map $H_{global}^{ang}$, and then superimpose the results of each area network to obtain *H*_*ma*_:

$$\begin{array}{c} Mask_{i}\left(m,n\right)=\{\begin{array}{cc} 1 & if\;\hat{H_{global}^{ang}}\left(m,n\right)\;\epsilon \;\varphi_{i},\\ 0 & otherwise, \end{array} \end{array}$$ (5)

where $\hat{H_{global}^{ang}}\left(m,n\right)$ denotes the predicted angle value of $H_{global}^{ang}$ in row m, column n.

Next, the fused features *H*_*ma*_ of each sub-network are obtained as:

$$\begin{array}{c} H_{ma}=\sum_{i}^{N}\;Mask_{i}\cdot H_{i}. \end{array}$$ (6)

**Fig 5:**
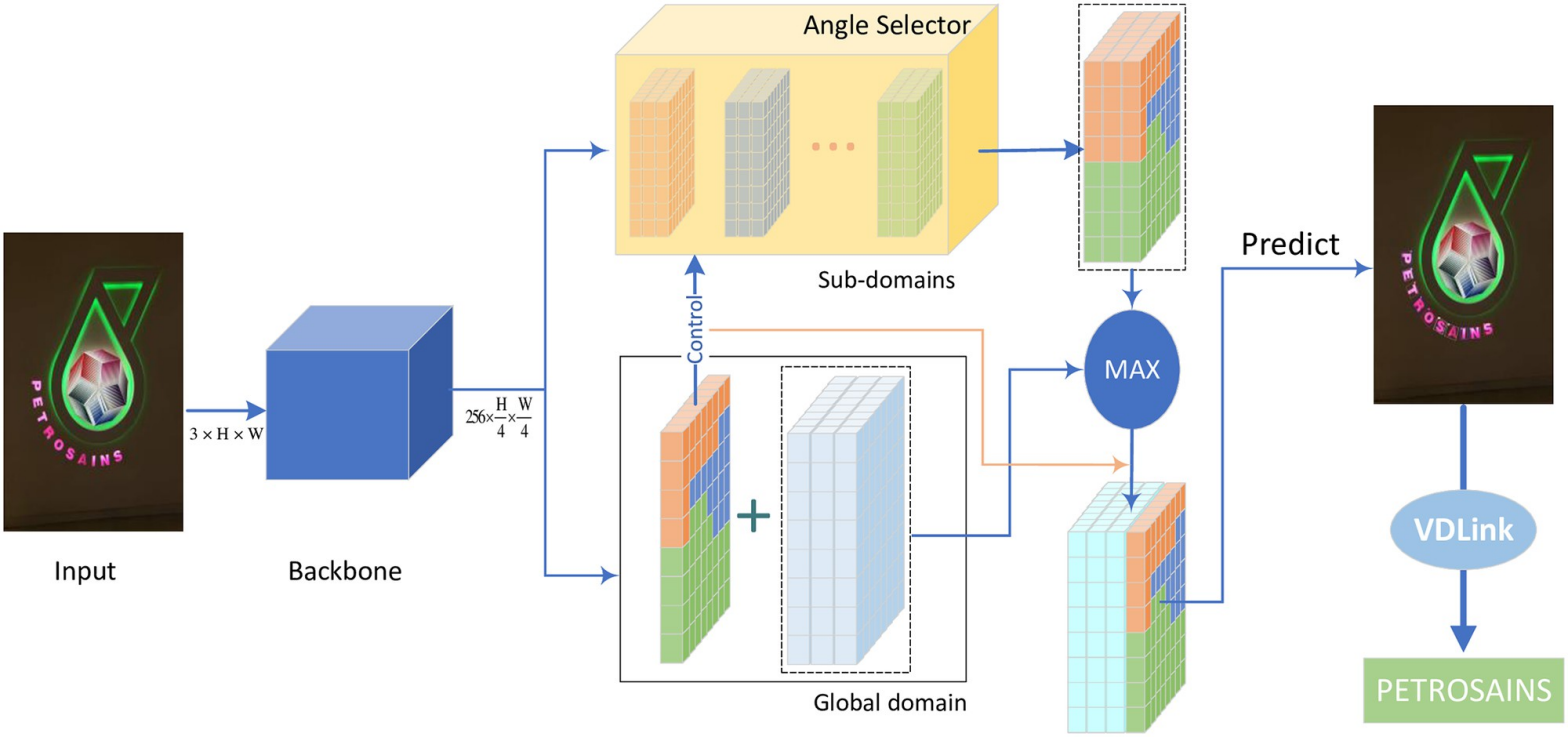
The framework of our proposed MA-CharNet. The global network and each sub-network share the Backbone, and use its 1/4 downsampled feature map as input. The angle selector selects the corresponding sub-network based on the angle feature map of the global network, and then further fuses this result with the output of the global network as the final output. Zoom in for better visualization. Reprinted from [19] under a CC BY license, with permission from IJDAR, original copyright 2020.

Angle Selector selects the corresponding sub-networks pixel by pixel according to the angle prediction map, which ensures the automatic fusion of each sub-network and the operational efficiency of MA-CharNet. However, the effectiveness of this method relies heavily on the performance of the angle prediction map. Considering that the global network *N*_*global*_ learns the character features of each angle domain, in order to avoid the wrong selection of sub-networks due to inaccurate angle prediction, this work further integrates the features of the fused sub-networks and the global network (the framework diagram of MA-CharNet is shown in Fig 5). In this study, the operation of averaging or maximizing *H*_*global*_ and *N*_*ma*_ was designed to alleviate the above problem, corresponding to Eqs (7) and (8), respectively:

$$\begin{array}{c} H_{fusion}\left(m,n\right)=\frac{1}{2}\cdot \left[H_{ma}\left(m,n\right)+H_{global}\left(m,n\right)\right], \end{array}$$ (7)

$$\begin{array}{c} H_{fusion}\left(m,n\right)=\text{max}\left\{H_{ma}\left(m,n\right),H_{global}\left(m,n\right)\right\}, \end{array}$$ (8)

where max{*H*_*ma*_(*m*, *n*), *H*_*global*_(*m*, *n*)} means take the larger of *H*_*ma*_(*m*, *n*) and *H*_*global*_(*m*, *n*).

Experiments have shown the validity of this idea(Table 4).

#### 3.1.3 Inference

Different from a single branch network, MA-CharNet integrates the output of multiple sub-networks according to the angle adaptive method, i.e., integrates the character features learned by the corresponding sub-networks in each angle domain.

More specifically, the processes of inference are as follows: first, inputting a image, backbone network extracts features from it, and feeds the 1/4 downsampled feature maps to the multi-heads of the global network and *N* sub-network; and then, the feature maps output by *N* sub-networks and the angle prediction maps generated by the global network are used as the input and control information of Angle Selector, respectively, to obtain the result *H*_*ma*_; finally, fuse *H*_*ma*_ and *H*_*global*_ to get *H*_*fusion*_, decode *H*_*fusion*_ to get the character recognition result.

In addition, after generating the prediction results of characters, they will be concatenated into words by the VDLink we designed.

### 3.2 Vector- and distance-based linking methods(VDLink)

Text recognition methods based on character detection usually require post-processing algorithms to connect characters into text sequences. The existing connection methods are usually based on people’s reading habits, that is, the characters are linked in the order from left to right. This rule can indeed better solve the connection of document text or general irregular text (as shown in Fig 7A), where the characters of such text have vertical or near-vertical horizontal lines in their central axis. However, in natural scenes, the left-to-right linking rule no longer applies because of the varying angles of the characters due to the shooting angle or the varying arrangement of the text itself (e.g., Fig 7B). Specifically, the reading sharing of the text should be related to the orientation of the characters.

**Fig 6:**
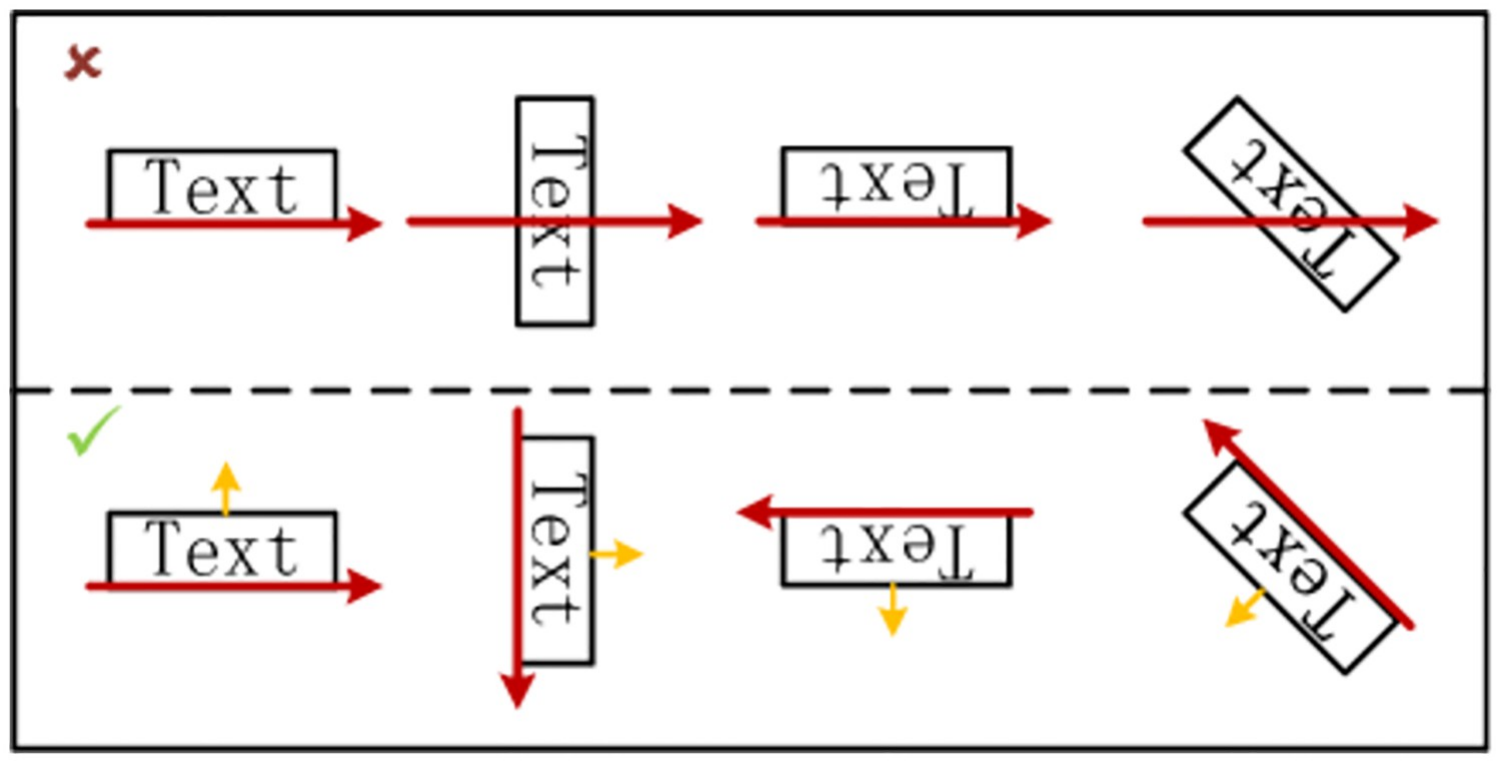
Comparison of two different link strategies.

**Fig 7:**
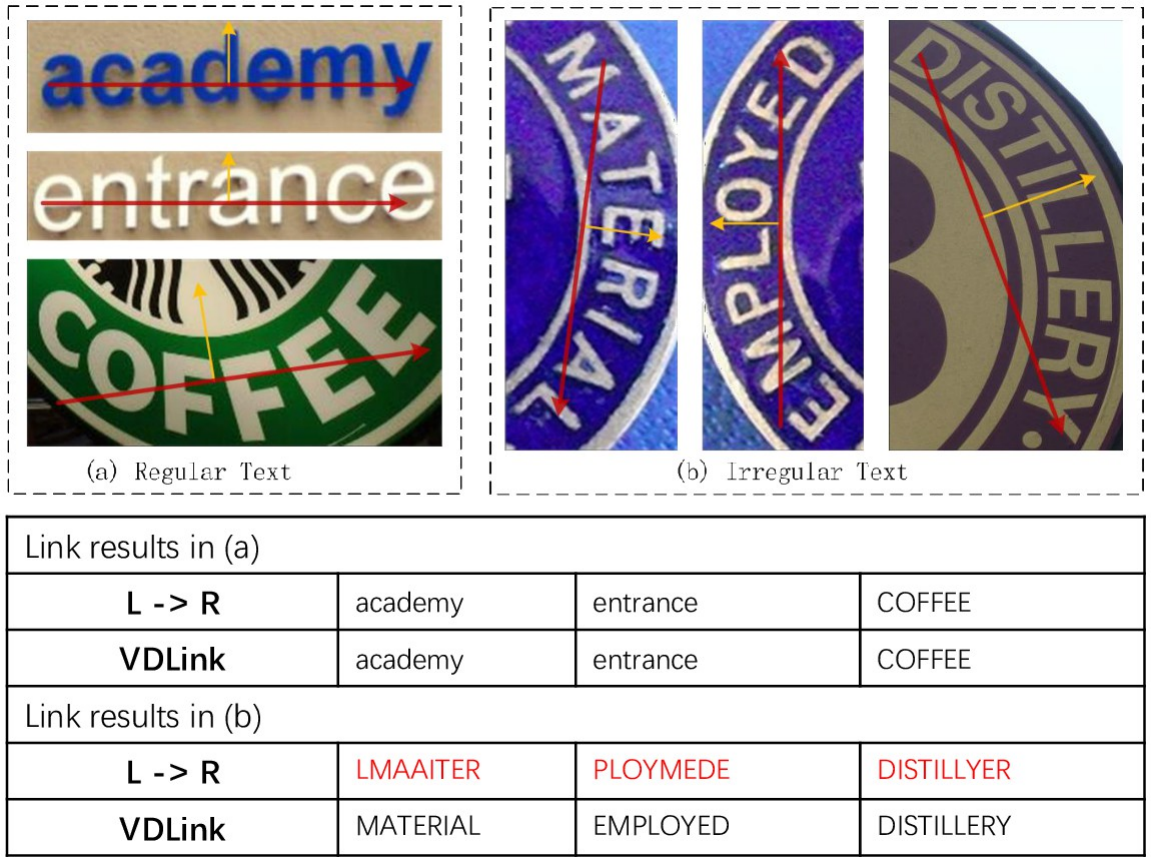
The result of using VDLink and left-to-right strategy on regular and irregular text. Reprinted from [19] under a CC BY license, with permission from IJDAR, original copyright 2020.

Unlike other text detection networks, MA-CharNet predicts the angle of individual characters, which provides sufficient reference information for determining the reading direction of the text. For the convenience of presentation, we denote the direction perpendicular to the central axis of the character upward as the direction of the character. As shown in Fig 6, the texts in different arrangements are listed. The yellow arrow indicates the direction of the character, and the red line indicates the reading direction of the text. The figure shows that the traditional left-to-right linking method is less robust, while the dynamic setting of the link direction to clockwise perpendicular to the direction of the characters can be well adapted to various arrangements of text. The rule also applies to general curved text, when the direction of the text takes the average direction of each character, the effect is shown in Fig 7B.

**Fig 8:**
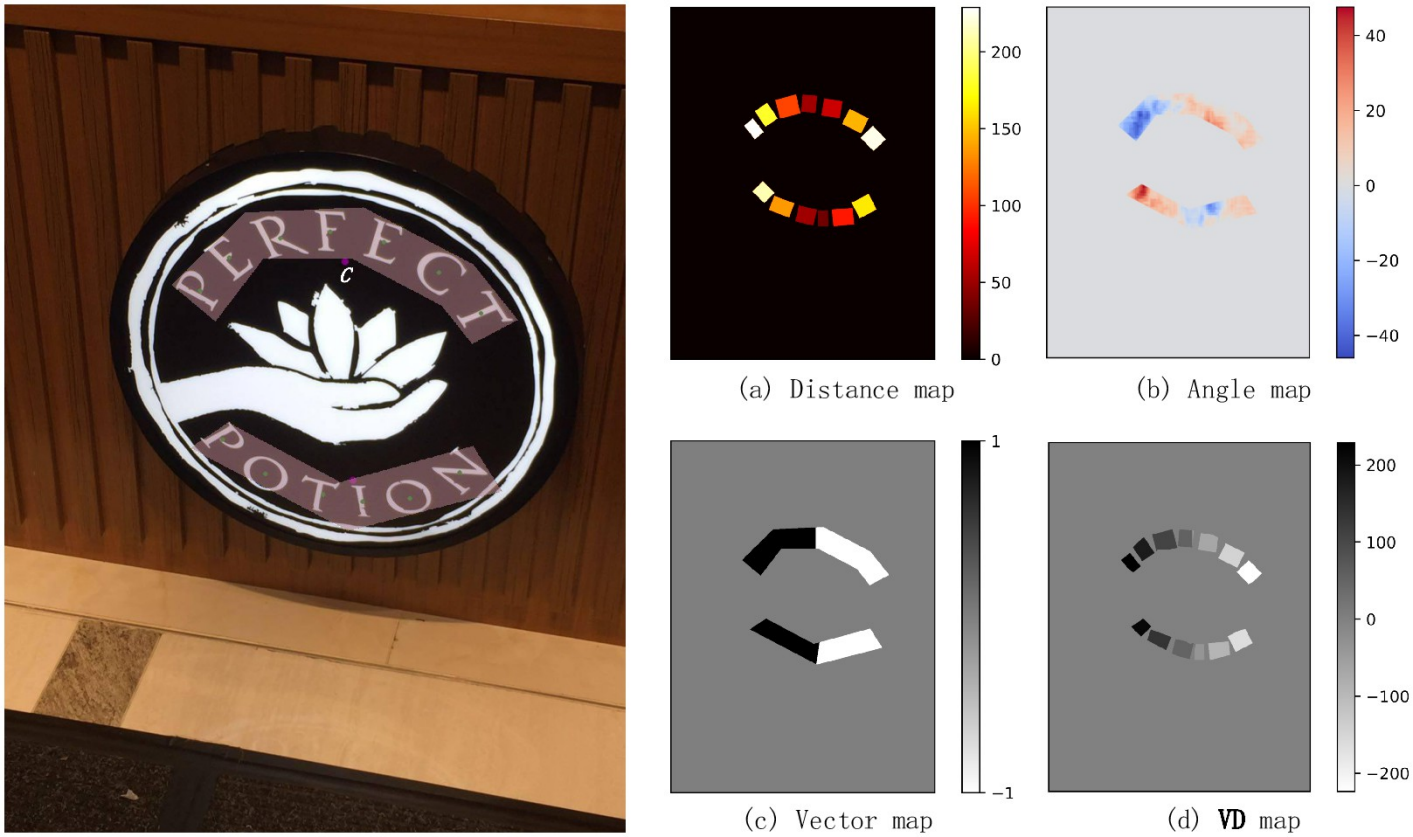
Diagram of VDLink’s processing. In the picture on the left, the green point is the center of the character detected by MA-CharNet, and the red point *C* is the centeroid of outer border. (a), (b), (c) and (d) denote the distance from the center of the character to the center of mass of the border, the angular prediction map of the character, the *v*_*i*_ value f and *VD*_*i*_ value of the character, respectively. Reprinted from [19] under a CC BY license, with permission from IJDAR, original copyright 2020.

As shown in Fig 8, the green point is the center of the character detected by MA-CharNet, and the red point *C* is the centeroid of outer border. The characters within the same outer border are recorded as *P*_1_, *P*_2_, …, *P*_*i*_, …, *P*_*n*_. The average angular prediction of the above sequence of characters is denoted as $\bar{\theta }$, and the vector from each character *P*_*i*_ to point *C* is denoted as $\vec{p_{i}}$. Then the vector $\vec{v}$, which determines the direction of the text link, satisfies:

$$\begin{array}{c} \vec{v}=\left(\text{cos}\bar{\theta },\text{sin}\bar{\theta }\right). \end{array}$$ (9)

The *v*_*i*_ representing the direction value of the character *P*_*i*_ is obtained by the operation of $\vec{v}$ and $\vec{p_{i}}$:

$$\begin{array}{c} v_{i}=\{\begin{array}{cc} 1 & if\;\vec{p_{i}}\cdot \vec{v}\;\geq 0,\\ -1 & otherwise. \end{array} \end{array}$$ (10)

Therefore, the comprehensive score *VD*_*i*_ of the character can be expressed as:

$$\begin{array}{c} VD_{i}=v_{i}*d_{i}, \end{array}$$ (11)

where *d*_*i*_ represents the distance from *P*_*i*_ to *C*.

Finally, the composite score *VD*_*i*_ of each character is sorted in descending order to get the sequence (…, *VD*_*m*_, *VD*_*n*_, …), then the character output sequence is (…, *P*_*m*_, *P*_*n*_, …).

## 4 Experiment

### 4.1 Datasets

#### Datasets for evaluation

MA-CharNet is designed to address the recognition of irregular text, so we will verify MA-CharNet on three public irregular data sets:

- **Total-Text**. [19] is an irregular data set containing 1500 training sets and 500 test sets, which contains most of vertical, horizontal, multi-oriented, and curved text. The format of the labels is given in word-level Polygon.
- **CTW1500**. [42] contains 1500 images, of which 1000 are used for training and 500 are used for testing. The test set contains 3,530 curve text instances. This data mainly contains horizontal and multi-oriented text. The dataset gives line-level annotations, which we validate at the word-level level.
- **CUTE80(CUTE)**. [43] contains 80 images and can cut out 288 pictures with only one text instance, a small amount of curved text, and perspective text and a blurred and variable background.

#### Datasets for training

Since datasets containing character-level annotations are more difficult to obtain, in addition to training on artificial datasets containing character labels, we also filtered some public datasets containing character-level annotations with higher quality.

- **SynthText** [44] consists of 800k images containing about 8 million horizontal, multi-oriented synthetic words. Each word is rendered into the scene and blending the words with the scene as much as possible. This dataset gives text line, word and character level annotations. It is generally used as a pre-training of the model.
- **ICDAR2013(IC13)** [45] contains 561 images, of which 420 are used for training and 141 for testing. The training set contains the annotations of the character set, and we randomly rotate the training set, and the rotated images are added to the training set.
- **ReCTS-25k** [46] contains 25k images, of which 20k are used for training and 5k are used for testing. Each character (containing Chinese and English) in this dataset is identified, and this work selects 10709 images containing only English and accurately annotated from the training set and added them to the training set.

Meanwhile, Total-Text dataset mentioned above does not contain character-level annotations, and 1168 images with character-level annotations were selected from the training set based on the segmentation map and word-level annotations. In addition, we generated 600k additional images with character-level annotations by generating characters with different colors and forms from 179 selected fonts and pasting them randomly on the background images, which are 8k images without text selected based on COCO-Text [47].

### 4.2 Implementation details

When training the global network *N*_*global*_ with artificial and partially real datasets, the ratio of each dataset fed to each batch is: SynthText: self: ICDAR2013: Rects: TotalText = 16 : 6 : 2 : 4 : 4, which remains the same when training the subnetwork *N*_*i*_. Only the dataset ICDAR2013, Rects, TotalText is rotated in the angle domain *φ*_*i*_ corresponding to the subnetwork *N*_*i*_. It should be noted that when training the sub-network, the backbone network Load the weights of the global network *N*_*global*_, and the weights of the backbone network are not updated, only the weight of the Ni detection head are updated. We train our model using 2 Tesla A100 GPUs with the image batch size of 64. We set the learning rate to 0.000125, decay the learning rate to half of the original every 3 epochs, and use Adam as the optimizer.

When training the global network *N*_*global*_, set *α* = *β* = *γ* = *δ* = 1 and set *α* = *β* = *γ* = 1, *δ* = 0 when optimizing the sub-network *N*_*i*_.

### 4.3 Results and analysis

**Fig 9:**
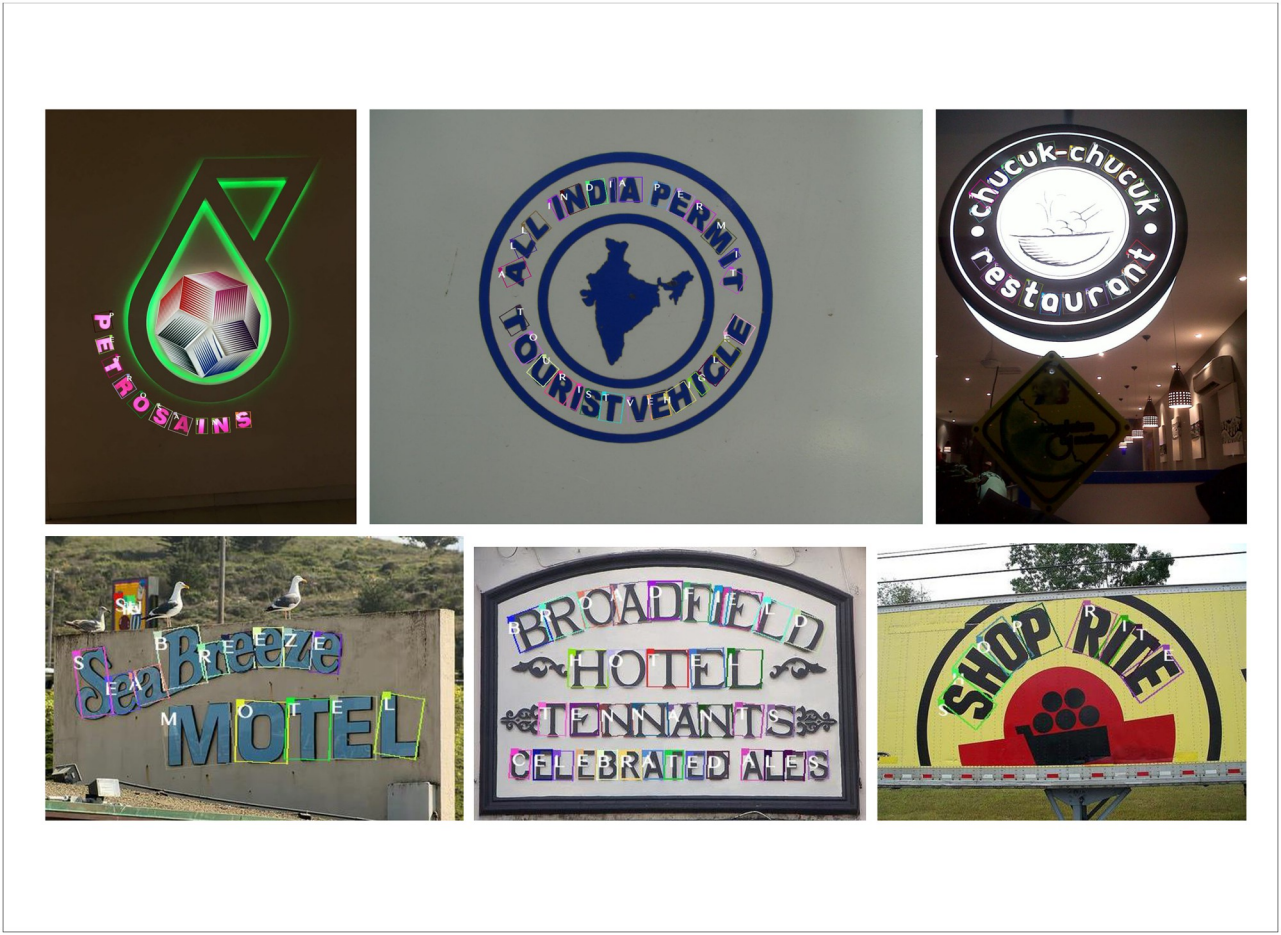
Qualitative results of MA-CharNet on irregular datasets. For better visualization, we do not show the given text outer border. Unlike other text recognition networks, MA-CharNet can recognize multiple texts in a single image at the same time. Reprinted from [19] under a CC BY license, with permission from IJDAR, original copyright 2020.

The recognition effect of MA-CharNet in different natural scenes is shown in Fig 9. The figure shows that our algorithm is robust to backgrounds, fonts, etc. Especially for characters with large inclination, it can also locate and recognize them accurately.

In addition, we compare with some current mainstream methods on three public datasets, and quantitatively analyze the results, as shown in Table 2. MA-CharNet achieves the best performance on TotalText and CTW, which are 2.4% and 2.7% higher than the current best algorithms, respectively. Since the main advantage of MA-CharNet is to deal with irregular text For CUTE80, this model is 2% lower than the current optimal algorithm. Nevertheless, the average performance of MA-CharNet on above three datasets is still 1.9% better than the existing algorithms.

**Table 2:** Irregular scene text recognize performance over public.

Method	Data	Annotations	Irregular Datasets			Avg
			*CUTE80*	*TOTAL*	*CTW*	
Parallel [7]	90K + ST	word	86.8	-	-	-
AON [48]	90K+ST	word	76.8	-	-	-
ACE [49]	90K	word	82.6	-	-	-
Xiao *et al*. [11]	90K+self	word+char	69.3	-	-	-
ESIR [19]	90K	word	83.3	-	-	-
ScRN [50]	90K+ST	word+char	87.5	-	-	-
SARv1 [14]	90K+ST+SA	word	83.3	-	-	-
SEED [10]	90K+ST	word	83.6	-	-	-
SCATTER [51]	90K+ST+SA	word	87.5	-	-	-
RobustScanner [9]	90K+ST	word	**90.3**	-	-	-
2D-CTC [12]	90K+ST	word	81.3	63.0	-	-
CTC [6]	90K	word	60.1	52.1	59.9	57.4
ASTER [21]	90K+ST	word	79.5	66.8	66.1	70.8
SARv2 [15]	90K+ST	word	83.3	68.6	73.9	75.3
SRN [22]	90K+ST	word	87.8	68.9	72.6	76.4
**Char-based(ours)**	ST+self+Real	char	88.1	69.2	74.4	77.2
**MA-CharNet(ours)**	ST+self+Real	char	88.3	**71.3**	**75.3**	**78.3**

The used training data and annotations are shown in columns ‘Data’ and ‘Annotations’, where ‘90K’, ’ST’, ’SA’, ’self’ refer to Synth90K dataset, SynthText dataset, SynthAdd dataset, self-made dataset, respectively. And ‘Real’ refer to the dataset we mentioned in Sec.4.1. ‘Avg’ denotes the average of the performance on the three irregular datasets. Note that all results are obtained without lexicon.

Note that dataset CUTE80 only consists of 288 word samples. Besides, it only contain a negligible proportion of irregular text samples [52]. However, MA-CharNet is designed for the recognition of irregular text, so our performance on CUTE80 is insufficient.

### 4.4 Ablation experiments

#### 4.4.1 Angle domain division number

In order to verify the effectiveness of the proposed multi-angle fusion method and its influence on speed, we conducted an ablation study on the number of angle domain divisions N. In this study, we set *φ* to $\left(-\frac{\pi }{2},\frac{\pi }{2}\right)$, i.e. $\zeta =-\frac{\pi }{2},\eta =\frac{\pi }{2}$ in Eq (3). In order to avoid side effects, set $\Delta \vartheta =\frac{\pi }{18}$ in Eq (4), so the range of the rotation angle of the characters in our data set is actually $\left(-\frac{5\pi }{9},\frac{5\pi }{9}\right)$.

We set the number of divided domains *N* to 1, 2, 3, 4 respectively, and the experimental results are shown in Table 3, When the angle is divided into 3 areas, the accuracy and speed can reach the best balance.

**Table 3:** Ablation study of the number of sub-domains.

Number of sub-domains(*N*)	Distribution by domain	Accuracy	FPS
1	$\left(-\frac{5\pi }{9},\frac{5\pi }{9}\right)$	69.2	27.4
2	$\left(-\frac{5\pi }{9},\frac{\pi }{18}\right)$ ,$\left(-\frac{\pi }{18},\frac{5\pi }{9}\right)$	70.7	23.7
3	$\left(-\frac{5\pi }{9},-\frac{\pi }{9}\right)$ ,$\left(-\frac{2\pi }{9},\frac{2\pi }{9}\right)$,$\left(\frac{\pi }{9},\frac{5\pi }{9}\right)$	71.3	21.1
4	$\left(-\frac{5\pi }{9},-\frac{7\pi }{36}\right)$ ,$\left(-\frac{11\pi }{36},\frac{1\pi }{18}\right)$,$\left(-\frac{\pi }{18},\frac{11\pi }{36}\right)$,$\left(\frac{7\pi }{36},\frac{5\pi }{9}\right)$	71.3	20.3

#### 4.4.2 Angle selector and fusion method

MA-CharNet learns the character features of each angle domain, and its Angle Selector module automatically selects the corresponding sub-networks. To verify the effectiveness of Angle Selector, we set up control experiments using only the global network, through Angle Selector fusion sub-networks, and further fusion with the global network after multi-angle fusion(as shown in Table 4).

**Table 4:** Ablation study of fusion method.

Fusion Method	Accuracy	FPS
global network	69.2	27.4
multi-domain fused^a^	71.1	21.2
mean(multi-domain, global)^b^	71.1	20.9
max(multi-domain, global)^c^	71.3	21.1

^a^ Eq (5),

^b^ Eq (7),

^c^ Eq (8)

The experimental results confirm the effectiveness of Angle Selector, which significantly improves the recognition performance despite a weak speed loss. Meanwhile, further fusion of the fused sub-networks with the global network brings a small performance improvement with little impact on speed.

#### 4.4.3 With or without VDLink

After MA-CharNet recognizes characters, it needs to link them into text sequences. To evaluate the effectiveness of the proposed component VDLink In this study, it is compared with the conventional left-to-right character linking method. Experiments(as shown in Table 5) show that the proposed VDLink has significant advantages.

**Table 5:** Ablation study of VDLink.

Linking Method	Accuracy
Left to Right	69.9
VDLink(ours)	**71.3**

## 5 Conclusion

We propose MA-CharNet, a novel framework for recognizing irregular text. Different sub-networks are used to learn character features in different angle domains separately, and then the accurate sub-network is selected autonomously by an adaptive angle selector (Angle Selector), which can well cope with the situation that characters in irregular text span a wide range of rotation angles. It achieves excellent performance while eliminating the tedious manual selection operation. The proposed curvature-adaptive character linking algorithm VDLink also provides a significant performance improvement over traditional character linking methods while incurring almost no computational overhead.

However, the accuracy of this model strongly depends on the accurate regression of the character angle, which directly affects whether a suitable character recognition sub-network can be selected for the detection of the target character. How to design a more efficient and accurate angle regressor for characters is the next focus of this work.

## Supporting information

S1 Appendix (ZIP)
